# Correction: Drug use and harm reduction in Afghanistan

**DOI:** 10.1186/1477-7517-2-20

**Published:** 2005-10-11

**Authors:** Catherine S Todd, Naqibullah Safi, Steffanie A Strathdee

**Affiliations:** 1 Division of International Health & Cross-Cultural Medicine, Department of Family & Preventive Medicine, University of California, San Diego, 9500 Gilman Drive, 0622, La Jolla, CA, 92093-0622, USA; 2 National HIV/AIDS Control Program, Ministry of Public Health, Massoud Road, Kabul, Afghanistan

The authors note that the above article [1] contained some inaccurate information. First, we have received clarification that no pilot program for methadone maintenance is currently planned, though there is agreement that such an activity is needed in Afghanistan.

Second, the name of the outreach and NEP program in Kabul is *Zandagi Nawin *located in the Dai Afghanan section of the city.

Last, the current locations for counseling and prevention activities are Kabul, Herat, Faizabad, Gardez, and Kandahar with Nejat Center (Kabul) and GTZ working as separate entities. GTZ is working with NGO partners KOR in Kabul and Faizabad, with Wadan in Gardez and Kandahar, and with SHRO in Herat. We apologize for any misrepresentation and thank Dr. Bayan Shairshah for bringing this to our attention.
